# Lack of association between single nucleotide polymorphisms in TCF7L2 and T2DM in the Chinese Yao population: A case-control study

**DOI:** 10.1097/MD.0000000000025326

**Published:** 2021-03-26

**Authors:** Shi-Yu Sun, Run-Ze Huang, Huang Huang, Ming-Qi Zhang, Hui-Lin Sun

**Affiliations:** a School of the Third Clinical Medicine, Guangzhou University of Chinese Medicine; b Department of Endocrinology, The First Affiliated Hospital of Guangdong Pharmaceutical University, Guangzhou, China.

**Keywords:** Chinese, single nucleotide polymorphisms, transcription factor 7-like 2 gene, type 2 diabetes mellitus

## Abstract

Single-nucleotide polymorphisms (SNPs) in the transcription factor 7-like 2 (TCF7L2) gene have been identified to be associated with the susceptibility to type 2 diabetes mellitus (T2DM) in various populations worldwide, but the results in Chinese are conflicting, and no data are available about the Liannan Yao population. Therefore, this study aimed to investigate the association of the TCF7L2 gene polymorphisms (rs12255372, rs7903146, rs7901695, rs11196205, and rs7895340) with T2DM in the Yao population living in the rural areas in the Liannan Yao Autonomous County.

This was a case-control study of 28 subjects with T2DM or prediabetes and 52 non-T2DM controls, all from the Chinese Yao population and recruited between January 2019 and June 2020. Patients with T2DM and prediabetes were grouped as the case group. The five SNPs (rs12255372, rs7903146, rs7901695, rs11196205, and rs7895340) were examined by polymerase chain reaction and direct genomic DNA sequencing in case and control groups.

The subjects in case group were older than the controls (55±14 vs 48 ± 15 years, *P* = .047), had higher FBG levels (9.31 ± 5.43 vs 4.09 ± 0.81, *P* < .001), higher TC (5.79 ± 1.29 vs 5.13 ± 1.18 mmol/L, *P* = .025), and higher triglycerides (2.94 ± 2.04 vs 1.86 ± 1.39 mmol/L, *P* = .003). The genotypic distribution for each of the SNPs was in agreement with the Hardy-Weinberg equilibrium. There were no statistically significant differences in the distributions of genotypes or alleles at all five SNPs of the TCF7L2 gene between the case and control groups (all *P* > .05).

TCF7L2 SNPs were not associated with T2DM in the Liannan Yao population.

## 1 Introduction

Type 2 diabetes mellitus (T2DM) is a common endocrine disorder characterized by variable degrees of insulin resistance and deficiency, resulting in hyperglycemia, which will result in renal, cardiovascular, and neurologic complications.^[1,2]^ T2DM is a complex trait that arises from the interplay of genetic and environmental factors.^[3]^ The number of diabetics worldwide was 415 million (9.1%) in 2015, and is predicted to increase to 642 million,^[4]^ leading to considerable economic and human burdens.^[5]^ In China, the prevalence of T2DM was < 1% in 1980, 5.5% in 2001, 9.7% in 2008, and 10.9% in 2013.^[6]^

A previous study by our group showed that the prevalence of T2DM in the Yao population living in the rural areas of Liannan Yao Autonomous County was 13.4%,^[7]^ higher than that of the general Chinese population. The Yao population living in rural areas has little ethnic inter-marriage with the Han population. They have their own language, culture, genetic background, lifestyle, and dietary habits. Identifying the causes underlying this high prevalence of T2DM has a profound significance to the prevention of the disease in the Liannan Yao population.

Several genetic variants are associated with T2DM among different ethnic populations. In 2006, Grant et al^[8]^ identified one microsatellite marker (DG10S478) within intron 3 of the transcription factor 7-like 2 gene (TCF7L2; formerly TCF4) associated with T2DM in Icelandic, Danish, and American cohorts. Five single-nucleotide polymorphisms (SNPs) (rs12255372, rs7903146, rs7901695, rs11196205, and rs7895340) were also found to be associated with T2DM in those three cohorts. Furthermore, it was shown that rs12255372 has a significant association with susceptibility to T2DM in the world population.^[9]^ On the other hand, the results of SNPs in TCF7L2 in the Chinese population yielded conflicting results.^[9–11]^ In addition, results about the SNPs of TCF7L2 in relation to T2DM in the Yao population of China are completely lacking.

Therefore, the present study aimed to investigate the association of the TCF7L2 gene polymorphisms (rs12255372, rs7903146, rs7901695, rs11196205, and rs7895340) with T2DM in the Yao population living in the rural areas in the Liannan Yao Autonomous County.

## 2 Materials and methods

### 2.1 Ethical approval

The present study was conducted in accordance with the guidelines of the Declaration of Helsinki. The protocol was approved by the Ethics Committee of the First Affiliated Hospital of Guangdong Pharmaceutical University (2016076). Written informed consent was obtained from each individual.

### 2.2 Study design and subjects

This was a case-control study of 28 subjects with T2DM or prediabetes and 52 non-T2DM controls, all from the Chinese Yao population and recruited between January 2019 and June 2020. The stratified cluster random sampling method was adopted to randomly select two townships in Liannan Yao Autonomous County, and three rural communities were randomly selected from the selected townships. The inclusion criteria were:

(1) > 18 years of age;
(2) Yao descent and living in the Liannan Yao Autonomous County for at least three generations; and
(3) unrelated individuals within three generations.

Patients with type 1 diabetes, hyperthyroidism, cancer, or immune diseases were excluded from the study.

The subjects with overnight fasting plasma glucose (FPG) levels > 7 mmol/L or who self-reported to be currently on diabetes treatments in the survey were included in the T2DM group, while people with FPG of 6.1 to 6.9 mmol/L or 2-h plasma glucose of 7.8 to 11.01 mmol/L were included in the prediabetes group, according to 1999 WHO criteria.^[12]^ Subjects with FPG levels < 6 mmol/L were included in the non-T2DM control group. The patients with T2DM and prediabetes were grouped as the case group. Blood glucose was measured by the Modified Folin Wu method.^[14]^

### 2.3 Data collection

All subjects underwent a physical examination (resting heart rate, systolic blood pressure (SBP), diastolic blood pressure (DBP), height, weight, waist circumference, and hip circumference) by experienced medical staff according to standard procedures. Body mass index was calculated as weight (kg) divided by height squared (m^2^).

Fasting blood samples (fasting at least 8 h) were collected into plain tubes and ethylenediaminetetraacetic acid tubes from all subjects and transported under cold conditions at prearranged intervals to the laboratory of the First Affiliated Hospital of Guangdong Pharmaceutical University. The plain tube samples were centrifuged at 4000 × g for 5 minute to separate the serum, which was used to analyze the biochemical parameters, including total cholesterol (TC), triglycerides (TG), high-density lipoprotein cholesterol, and uric acid. TC, TG, and high-density lipoprotein cholesterol were quantitated by classical chemical methods,^[15]^ while uric acid assessment used the uricase-peroxidase coupling method.^[16]^

### 2.4 Polymerase chain reaction (PCR) amplification and genotyping

The ethylenediaminetetraacetic acid samples were used for DNA analysis. DNA was extracted from the peripheral blood using centrifugal columns and amplified using PCR as follows. The reaction system (40 μl) included ddH2O (24.6 μl), dNTP (3.2 μl), 10x Taq Buffer (4 μl), forward and reverse primers (2 μl each), Ex Taq DNA Polymerase (0.2 μl) and DNA template (4 μl) (Takara Bio, USA), as directed by the manufacturer. PCR was performed at 94°C for 3 minutes, 94°C for 30 seconds and 72°C for 40 seconds for 30 to 35 cycles, followed by final extension at 72°C for 5 minutes. Samples were analyzed by 1.0% agarose gel for electrophoresis, and imaging used the UV gel imager.

For genotyping, each PCR amplified product was attributed a serial number for gene sequencing. The Lasergene SeqMan Pro software (DNASTAR, Inc., Madison, WI, USA) was used to perform genotyping of the corresponding SNP on the gene sequence. According to the NCBI gene database, the SNPs had the following genotypes: rs7901695, three genotypes (homozygous TT, heterozygous TC, and wild type homozygous CC); rs7903146, three genotypes (homozygous CC, heterozygous CT, and wild homozygous TT); rs7895340, three genotypes (homozygous GG, heterozygous GA, and wild type homozygous AA); rs11196205, three genotypes (homozygous GG, heterozygous GC, and wild homozygous CC). In addition, rs12255372 (G/T), rs7903146 (C/T), rs7901695 (T/C), rs11196205 (G/C), and rs7895340 (G/A) SNPs of the TCF7L2 gene were also examined. Those SNPs were those found to be carrying a higher risk of T2DM in other ethnic groups.^[8]^ The primer sets for PCR are listed as Table 1. The final PCR products were analyzed on 2% agarose gel stained with ethidium bromide before gene sequencing was carried out by the Department of Medical Genetics of Southern Medical University.

**Table 1:** Primer sequences for each SNP.

SNPs	PCR Primers Sequences
rs12255372 (G>T)	F: 5’-GGAAACTAAGGCGTGAGGGAC-3’
	R: 5’- GTGGATTCTGGGCATGCTAA -3’
rs7903146 (C>T)	F: 5’-CACCGAGTTTAGCCCAGGTTC-3’
	R: 5’-AAGTGCCCAAGCTTCTCAGTC-3’
rs7901695 (T>C)	F: 5’-TGCAGTCATCCACACCCTCTA-3’
	R: 5’-ATGACCCAGCAATTCCACTCA-3’
rs11196205 (G>C)	F: 5’-AGCCCATAATCTCACCACTCG-3’
	R: 5’-GTTAAGGGCATGTCTCTCTCCA-3’
rs7895340 (G>A)	F: 5’- TCAGGGACAGTGCATAGGTGT -3’
	R: 5’- ACTCTGAACCACTGCCAGGAT -3’

PCR = polymerase chain reaction, SNPs **=** single-nucleotide polymorphisms.

### 2.5 Statistical analysis

SPSS 20.0 for Windows (IBM Corp, Armonk, NY, USA) was used for all analyses. Continuous data were presented as means ± standard deviation and analyzed using Student *t*-test. Categorical data were presented as n (%) and analyzed using the chi-square test and Fisher exact test. The Hardy-Weinberg equilibrium was tested using the chi-square test. Logistic regression analysis with odds ratios and 95% confidence intervals was used to assess the contribution of major risk factors. Two-sided *P*-values < .05 were considered statistically significant.

## 3 Results

### 3.1 Characteristics of the participants

Based on the above eligibility criteria, a total of 89 people were included, and 9 non-third-generation direct relatives were excluded. Table 2 shows the demographic and clinical characteristics of the subjects. The subjects in case group were older than the controls (55 ± 14 vs 48 ± 15 years, *P* = .047), had higher FBG levels (9.31 ± 5.43 vs 4.09 ± 0.81, *P* < .001), higher TC (5.79 ± 1.29 vs 5.13 ± 1.18mmol/L, *P* = .025), and higher TG (2.94 ± 2.04 vs 1.86 ± 1.39 mmol/L, *P* = .003). There were no significant differences between the two groups for the other variables (all *P* > .05).

**Table 2:** Demographic and clinical characteristics of the subjects.

Characteristics	Case (n = 28)	Control (n = 52)	*P*
Age (yr)	55 ± 13.5	48 ± 14.67	.047
Sex (male/female)	16/12	21/31	.152
BMI (Kg/m^2^)	24.25 ± 4.19	23.55 ± 5.43	.728
WHR	0.88 ± 0.07	0.86 ± 0.05	.070
SBP (mm Hg)	138.71 ± 21.54	130.42 ± 19.02	.078
DBP (mm Hg)	87.29 ± 12.14	84.23 ± 11.98	.395
FBG (mmol/L)	9.31 ± 5.43	4.09 ± 0.81	<.001
TC (mmol/L)	5.79 ± 1.29	5.13 ± 1.18	.025
TG (mmol/L)	2.94 ± 2.04	1.86 ± 1.39	.003
HDL (mmol/L)	1.56 ± 0.34	1.53 ± 0.33	.692
LDL (mmol/L)	2.89 ± 1.08	2.75 ± 1.07	.593
UA (mmol/L)	368.64 ± 148.94	368.58 ± 101.54	.632
Cr (μmol/L)	73.5 ± 21.11	67.42 ± 13.34	.175

Continuous variables are expressed as mean ± SD. BMI = body mass index, Cr = creatinine, DBP = diastolic blood pressure, HDL = high-density lipoprotein, LDL = low-density lipoprotein, PFG = fasting plasma glucose, SBP = systolic blood pressure, TC = total cholesterol, TG = triglyceride, UA = uric acid, WHR = waist to hip ratio.

### 3.2 Comparison of genotype and alleles between two groups

Table 3 shows the distribution of the genotypes and alleles for the five SNPs of the TCF7L2 gene in the Yao population. The genotypic distribution for each of the SNPs was in agreement with the predicted Hardy-Weinberg equilibrium values. There were no statistically significant differences in the distributions of either genotypes or alleles at all five sites of the TCF7L2 gene between the case and control (all *P* > .05) groups.

**Table 3:** Genotype and allele distributions in case and control groups.

Genotype	Case	Control	χ^2^	*P*	OR (95%CI)
rs12255372 (G/T)					
GG	26	46	0.391	.706	
GT	2	6			
TT	0	0			
G	54	98	0.370	.714	1.023 (0.955–1.097)
T	2	6			0.619 (0.129–2.967)
rs7903146 (C/T)					
CC	25	46	2.210	.409	
CT	2	6			
TT	1	0			
C	52	98	0.117	>.99	0.985 (0.903–1.075)
T	4	6			1.238 (0.365–4.205)
rs7901695 (T/C)					
TT	25	46	0.012	>.99	
TC	3	6			
CC	0	0			
T	53	98	0.012	>.99	1.004 (0.929–1.086)
C	3	6			0.929 (0.241–3.572)
rs11196205 (G/C)					
GG	26	46	0.391	.706	
GC	2	6			
CC	0	0			
G	54	98	0.370	0.714	1.023 (0.955–1.097)
C	2	6			0.619 (0.129–2.967)
rs7895340 (G/A)					
GG	26	46	0.391	0.706	
GA	2	6			
AA	0	0			
G	54	98	0.370	0.714	1.023 (0.955–1.097)
A	2	6			0.619 (0.129–2.967)

CI = confidence interval, OR = odds ratio.

## 4 Discussion and conclusions

SNPs in TCF7L2 have been identified to be associated with the susceptibility to T2DM in various populations worldwide,^[8,10,13–16]^ but no data are available about the Liannan Yao population. Therefore, this study aimed to investigate the association of the TCF7L2 gene polymorphisms with T2DM in the Yao population living in the rural areas in the Liannan Yao Autonomous County. The results suggested that the TCF7L2 SNPs were not associated with T2DM in the Liannan Yao population.

As the only hypoglycemic hormone in the body, insulin is secreted by pancreatic β-cell. The defective β-cell mass and impaired β-cell function are considered as the main reason for the impaired insulin secretion, which could lead to impaired glucose tolerance and induce T2DM eventually.^[17]^ The TCF7L2 gene encodes a significant transcription factor of the Wnt signaling pathway, which is both necessary and sufficient for islet β-cell proliferation, differentiation, and endocrine function.^[18]^ Shu et al.^[19]^ reported that TCF7L2 might be an important regulator of β-cell function and survival, and a low-expression of TCF7L2 in β-cells from carriers of at-risk alleles might play an important role in the progression of defective insulin secretion and T2DM.^[20,21]^ The glucagon-like peptide-1 (GLP-1) is a hormone secreted by intestinal L cells that can promote the proliferation of pancreatic islets and stimulate insulin secretion. Normally, the TCF7L2 gene can regulate the expression of GLP-1 to maintain insulin secretion through the Wnt signaling pathway. Schafer et al.^[22]^ confirmed that variants of TCF7L2 could specifically impair GLP-1-induced insulin secretion and suggested that TCF7L2 polymorphisms might confer an increased risk of T2DM. Though the precise pathogenic mechanism of TCF7L2 polymorphisms in the pathogenesis of T2DM has yet to be elaborated thoroughly, its impact on β-cells is accepted by scholars worldwide.^[20,21]^

Owing to the human genome project, several genes were found to be associated with the prevalence of T2DM. Among them, the TCF7L2 gene might be the most influential genes conferring genetic susceptibility to T2DM in humans. The first breakthrough of the association between genetic polymorphisms of the TCF7L2 gene and T2DM was reported by Grant et al.,^[8]^ who found that a microsatellite (DG10S478) within intron 3 of the TCF7L2 gene was associated with T2DM (*P* = 2.1 × 10^–9^) in Icelandic individuals, and this association was replicated in a Danish cohort (*P* = 0.005) and in a cohort from the United States of America (*P* = 3.3 × 10^−9^). Since this initial study was published, other studies examined the association between SNPs in the TCF7L2 gene and T2DM, including the United Kingdom,^[13]^ France,^[14]^ Spain,^[15]^ China,^[10]^ and the United States of America.^[16]^ Though a consensus was reached about the association between the TCF7L2 gene and T2DM, the relevant locus remains controversial. In a study by Hayashi et al.,^[23]^ the four investigated SNPs (rs12255372, rs7903146, rs7901695, and rs11196205) were significantly associated with T2DM in the Japanese population, among which rs12255372 showed the strongest association (OR = 1.7, 95%CI: 1.20–2.41, *P* = .0024), but the frequency of the minor allele in the Japanese population was lower than that in a European Caucasian population. Similarly, Miyake et al^[24]^ found that rs7903146, rs11196205, and rs12255372 might contribute to the genetic susceptibility to T2DM in the Japanese population, while neither rs11196218 nor rs290487 showed a significant association.

On the other hand, Chang et al^[25]^ did not detect any association of T2DM with rs7903146 and rs12255372 but confirmed that rs290487 was associated with T2DM. Dou et al.^[26]^ indicated that there was no evidence that the rs12255372 and rs290487 SNPs increased the T2DM risk (OR = 1.77, 95%CI = 0.88–3.56; OR = 1.08, 95%CI = 0.93–1.25) in a Chinese population. In a case-control study and meta-analysis, Ren et al^[27]^ found no association between rs290487 and T2DM in the Chinese population.

In the present study, no significant differences were observed in either the genotypic frequencies or allelic frequency of rs12255372 and rs290487 between cases and the controls. Though no significant differences were found in the TCF7L2 genotypes and alleles, some factors were still different between the two groups. Dyslipidemia observed in patients with diabetes is associated with high insulin resistance,^[28]^ and insulin resistance can cause an alteration in lipid metabolism.^[29–31]^ In accordance with previous studies, this study indicated that dyslipidemia might play an important role in the pathophysiology of T2DM.

There were several limitations to this study. First, the conclusion might not represent the whole Yao population in China because of the relatively small sample size. Secondly, the case group included patients with prediabetes, which might lead to selection bias; in addition, other potential confounders were not taken into consideration, e.g., a family history of diabetes, duration of diabetes, lifestyle (sedentary vs. active), cigarette smoking, alcohol use, dietary habits, medication use and comorbidities such as chronic obstructive pulmonary disease (COPD). Thirdly, only five SNPs were examined, and there is a possibility that other TCF7L2 SNPs might exist. There is also the possibility that SNPs in other genes might be responsible for the high prevalence of T2DM in the Yao population, or maybe a specific gene-environment interaction. Fourthly, although controls and diabetics had comparable body mass index values, we did not measure visceral obesity, which could be informative. Finally, the case and control groups significantly differed in age, which might by itself explain the differences observed. Additional studies are still necessary to confirm our findings.

In conclusion, the results of the present study suggested that the TCF7L2 SNPs were not associated with T2DM in the Liannan Yao population. In the future, a systematic study of other SNPs in or adjacent to TCF7L2 in a larger case–control study in the Liannan Yao population are warranted to evaluate the role of this gene in the genetic predisposition to type 2 diabetes in the Liannan Yao population.

## Acknowledgments

We acknowledge the help of the People's Government of Liannan County and the People's Hospital of Liannan County in Guangdong for their assistance in sample collection and processing. We thank all the participants in the study. We thank the First Affiliated Hospital of Guangdong Pharmaceutical University and Guangdong Huamei Zhongyuan Biotechnology Co., Ltd.

## Author contributions

Shi-Yu Sun analyzed and interpreted the data, and was a major contributor in writing the manuscript. Run-Ze Huang was secondary contributors in analyzing data, Huang Huang and Ming-Qi Zhang were secondary contributors in writing the manuscript. Hui-Lin Sun designed and guided this project. All authors read and approved the final manuscript.

**Investigation:** Ming-Qi Zhang.

**Methodology:** Shi-Yu Sun, Run-Ze Huang.

**Visualization:** Huang Huang.

**Writing – original draft:** Shi-Yu Sun.

**Writing – review & editing:** Shi-Yu Sun, Hui- Lin Sun.
